# ClinGen Allele Registry links information about genetic variants

**DOI:** 10.1002/humu.23637

**Published:** 2018-10-11

**Authors:** Piotr Pawliczek, Ronak Y. Patel, Lillian R. Ashmore, Andrew R. Jackson, Chris Bizon, Tristan Nelson, Bradford Powell, Robert R. Freimuth, Natasha Strande, Neethu Shah, Sameer Paithankar, Matt W. Wright, Selina Dwight, Jimmy Zhen, Melissa Landrum, Peter McGarvey, Larry Babb, Sharon E. Plon, Aleksandar Milosavljevic, on behalf of the Clinical Genome (ClinGen) Resource

**Affiliations:** ^1^ Department of Molecular and Human Genetics Baylor College of Medicine Houston Texas; ^2^ Renaissance Computing Institute University of North Carolina Chapel Hill North Carolina; ^3^ Geisinger's Autism and Developmental Medicine Lewisburg Pennsylvania; ^4^ Department of Genetics University of North Carolina Chapel Hill North Carolina; ^5^ Department of Health Sciences Research Mayo Clinic Rochester Minnesota; ^6^ Department of Biomedical Data Sciences Stanford University School of Medicine Palo Alto California; ^7^ National Center for Biotechnology Information National Institutes of Health Bethesda Maryland; ^8^ Innovation Center for Biomedical Informatics Georgetown University Medical Center Washington District of Columbia; ^9^ Sunquest Information Systems Company Boston Massachusetts; ^10^ Department of Pediatrics Baylor College of Medicine Houston Texas

**Keywords:** HGVS representation, linked data, pathogenicity of genetic variants, variant centric resources, variant identifiers

## Abstract

Effective exchange of information about genetic variants is currently hampered by the lack of readily available globally unique variant identifiers that would enable aggregation of information from different sources. The ClinGen Allele Registry addresses this problem by providing (1) globally unique “canonical” variant identifiers (CAids) on demand, either individually or in large batches; (2) access to variant‐identifying information in a searchable Registry; (3) links to allele‐related records in many commonly used databases; and (4) services for adding links to information about registered variants in external sources. A core element of the Registry is a canonicalization service, implemented using in‐memory sequence alignment‐based index, which groups variant identifiers denoting the same nucleotide variant and assigns unique and dereferenceable CAids. More than 650 million distinct variants are currently registered, including those from gnomAD, ExAC, dbSNP, and ClinVar, including a small number of variants registered by Registry users. The Registry is accessible both via a web interface and programmatically via well‐documented Hypertext Transfer Protocol (HTTP) Representational State Transfer Application Programming Interface (REST‐APIs). For programmatic interoperability, the Registry content is accessible in the JavaScript Object Notation for Linked Data (JSON‐LD) format. We present several use cases and demonstrate how the linked information may provide raw material for reasoning about variant's pathogenicity.

## 1 INTRODUCTION

Genome research and genomic medicine both depend on the community's ability to effectively exchange and aggregate information about genetic variants. Our immediate motivation came from the need for unique variant identifiers, particularly for variants not previously registered in ClinVar and for the variants undergoing pathogenicity assessment by the ClinGen‐supported expert panels. Assessment of a variant's pathogenicity frequently requires information derived from literature, population sequencing databases, high‐throughput experiments, curated databases such as ClinVar (Landrum et al., 2016), and a growing number of other sources. More generally, the increasing pace of data accumulation and the growing diversity of resources and variant nomenclatures are challenging the consumers’ ability to effectively Find and Access (the “F” and “A” in “FAIR” standard, respectively; Wilkinson et al., 2016), information about an allele with any certainty that the same allele is being referenced. One key aspect of the problem is the lack of globally unique variant identifiers that would enable the aggregation and connection of information from different sources about the same variant. Non‐Single Nucleotide Polymorphism (SNP) variants such as indels may be represented in many different ways, each corresponding to a different human genome variation society (HGVS) expression. Although the problem may be solved in principle by defining “canonical” HGVS expressions and standardizing on a set or reference sequences, practical implementation of this concept is challenging as it requires reconciling “canonical” expressions across a multiplicity of transcript sequences frequently used in clinical genetics.

To overcome the limitations of currently available systems and to address the data aggregation problem at scale, we developed the Clinical Genome resource (ClinGen) Allele Registry. The Registry provides globally unique “canonical” variant identifiers (the “CAids”) on demand via web (UI or API) services. A user‐friendly web interface provides various ways to query existing and register new variants. The canonical identifiers may be obtained either individually or in high volume via web APIs to meet the registration needs of external databases of any size. The canonicalization service is provided using a custom in‐memory index that is based on the alignment of hundreds of thousands of transcripts and genomic sequences. For each canonical identifier, there is typically a multiplicity of HGVS notations for the same variant in the context of common genomic and transcript reference sequences. The Registry also provides web UIs to support identification and registration of individual variants from partial descriptions present in the literature, genetic test reports, or in other sources.

Another important Registry feature is the support for linking to information about registered variants in external sources frequently used by clinicians, diagnostic laboratories, and researchers. The Registry currently links to major resources such as gnomAD, ExAC (Lek et al., 2016), dbSNP (Sherry et al., 2001), MyVariant.info (Xin et al., 2016), COSMIC (Forbes et al., 2015, 2017), and ClinVar (Landrum et al., 2016). Moreover, the Registry also supports on‐demand registration of links to additional layers of variant information available from any number of external sources, small or large. The on‐demand registration of millions of new variants per request via the APIs is designed to meet the needs of all global genome sequencing efforts that generate germline and somatic variants, of high‐throughput in vitro mutagenesis experiments that generate information about functional effects of variants that previously may not have been seen in humans, and even of computational prediction projects that provide information about variants that are yet to be observed in humans or in vitro.

Here, we describe the development and implementation of the ClinGen Allele Registry, its content, and key services. We also summarize sources that use Registry identifiers in their systems. Additionally, we demonstrate both manual access via user‐friendly web interfaces and programmatic access via the REST‐ APIs. Finally, we show how the information linked by the Registry may be “mined” for information about variants’ pathogenicity.

## 2 METHODS

### 2.1 Overall design and data flow

Allele Registry services (available at https://reg.clinicalgenome.org) facilitate the linking of variant information by providing globally unique “canonical” variant identifiers on demand. To identify groups of equivalent variant representations and assign them a unique canonical identifier, the Registry normalizes variant representations within individual reference sequences and maps them across known reference sequences, including different versions of genome assemblies, assembled loci, and transcripts. The amino acid sequences are not stored or aligned explicitly—they are computed on‐the‐fly from protein‐coding transcript sequences. The registry provides search functionality to help identify a variant using common types of variant‐identifying information. A service is available for registering new variants, either individually or in batches. Registration requires a login that is instantly created on demand.

**Figure 1:**
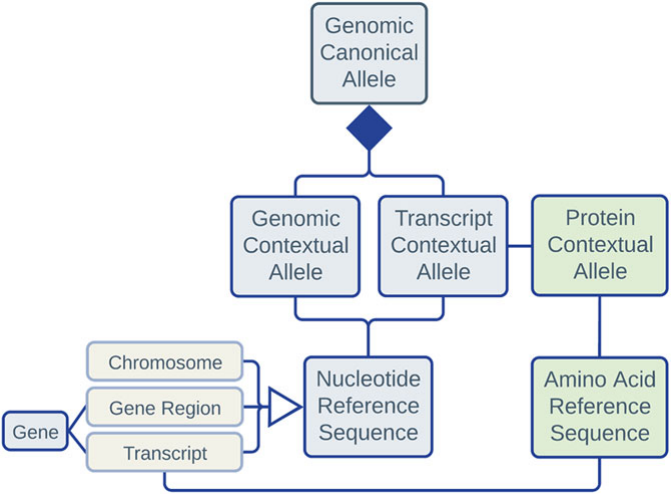
Conceptual model of Allele Registry entities based on the Allele Model developed by ClinGen Data Model Working Group

The Registry is based on the Allele Model developed by the ClinGen Data Model Working Group (**Figure** 1, https://dataexchange.clinicalgenome.org/allele/master/index.html). Briefly, a contextual allele is defined in the context of a specific reference sequence. All contextual alleles corresponding to a same variant are associated with the same canonical allele. Each canonical allele has its own “CAid” number (e.g., CA321211) and persistent and dereferenceable universal resource identifier (URI) (e.g., https://reg.genome.network/allele/CA321211) that serves as a Globally Unique Identifier (GUID) for the variant. The maximal nucleotide (transcript or genomic) allele size is 10,000 bp, a cutoff selected based on tradeoff between the need to accommodate large alleles and the need to efficiently store and process them. The Registry also allows for “complex” alleles including haplotypes (e.g., CA033016, a haplotype allele that is also present in ClinVar: NM_000402.4:c.[292G > A;466A > G]); however, it treats each haplotype as a single variant, not modeling individual variants that constitute a haplotype explicitly. The nucleotide (genomic and transcript) and protein variants are treated as different types of entities that may be joined by a one‐to‐many relationship (every transcript variantcausing at most one amino acid sequence change, whereas each amino acid sequence variant corresponding to possibly one or more transcript variants).

The Registry's backend is implemented in C++ as a multithreaded HTTP server providing publicly available REST‐API. The services are highly optimized for query and registration of tens of thousands of variants per second. Responses are returned according to Linked Data standards in RDF‐serializable JSON‐LD format or as annotated variant call format (VCF) files. The UIs are implemented in Ruby as Genboree plugins. Registration of new variants and of new sources of information about them requires authentication and authorization, whereas a query does not. Any user is currently authorized to register variants upon creating an account, a process that can be completed in less than 1 min. Although currently not implemented, Allele Registry will support widely used open authentication systems in future.

**Figure 2:**
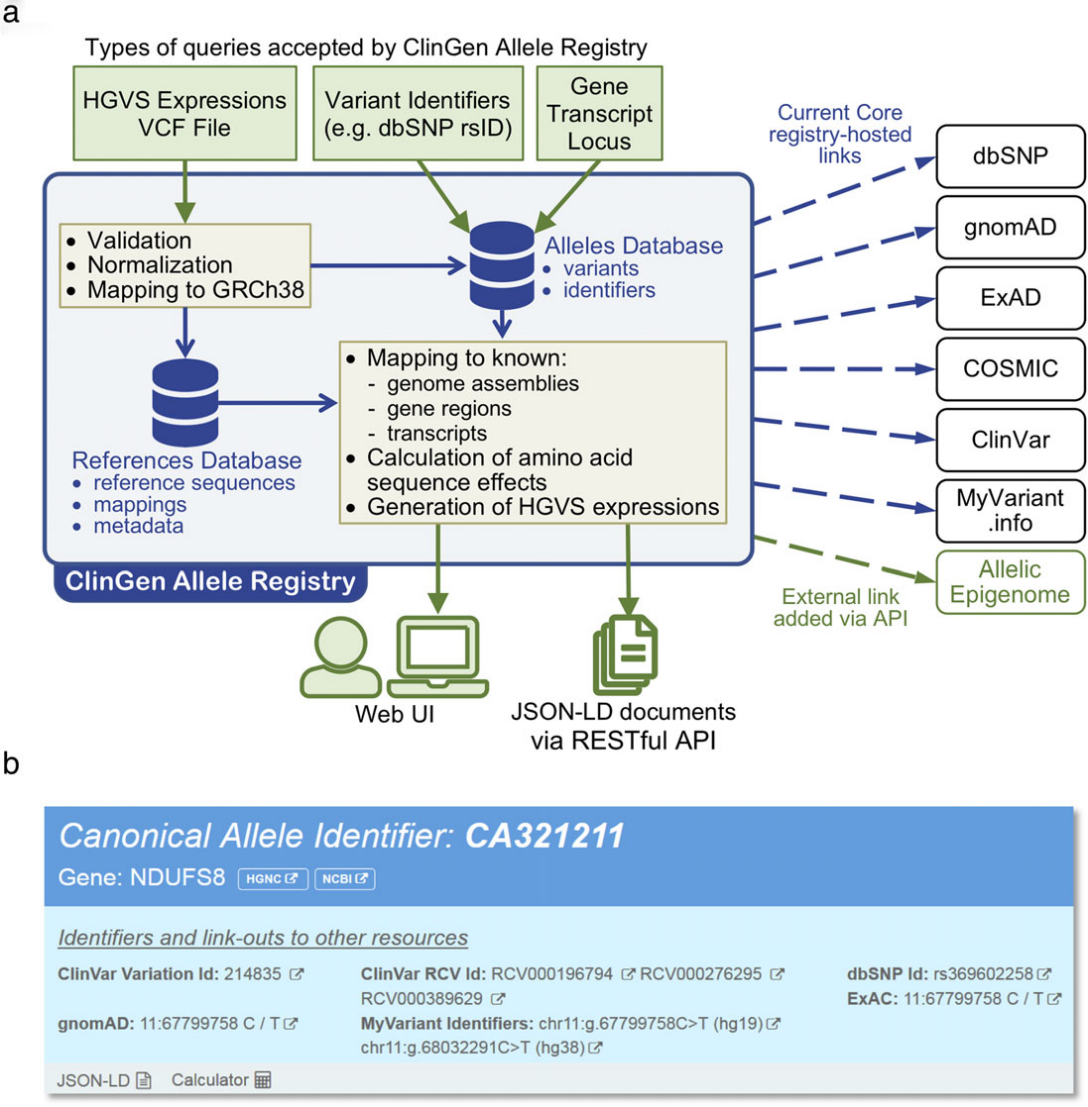
(a) Design and workflow of ClinGen Allele Registry. (b) Screenshot of current core registry‐hosted links for a typical variant in the user interface

The overall data flow of the Registry is summarized in Figure 2. Input variant descriptions are parsed, validated, and represented internally as series of contextual alleles residing on specific reference sequences (as detailed below in Section 2.2). The normalization step produces a unique variant representation in the context of a specific reference sequence (Section 2.3). The canonicalization step calculates the variant's representation, independent of sequence context, using a sequence‐alignment based index (Section 2.4). The Registry supports both the retrieval of previously registered canonical variants and the registration of new variants. To support the data flow and provide these services, the Registry utilizes two internal databases, the reference database consisting of reference nucleotide (genomic and transcript) sequence alignments (Section 2.5) and an allele database (Section 2.6). A key feature of the Registry is its set of tools and services that support the sharing of links to information about registered variants in sources external to the Registry (Section 2.7).

### 2.2 Parsing and validation

The Registry parser takes as input either VCF representations or HGVS expressions representing the following four types of nucleotide variants: single nucleotide variants (SNVs), insertions, deletions, and indels (illustrated in Table 1). A wide diversity of variants and their HGVS representations are supported, including variants defined in transcripts’ intronic regions. All parsed positions and reference alleles are validated by comparison against reference sequences. If the validity check fails (e.g., due to a mismatched reference allele), the registry stops further processing of the variant and informs the user about the error. Successful parsing and validation produces the following four attributes identifying a contextual allele:  (1) reference sequence, (2) position in the reference sequence, (3) the reference allele, and (4) the alternate allele.

**Table 1:** Types of variants within the Registry

	Reference and alternated sequences	Region of alteration	Alternate allele	Reference allele
SNV	ACTGTCGTG ACTGACGTG	[4, 5]	A	T
Insertion a	ACTG__TCGTG ACTGACTCGTG	[4, 4]	AC	(Empty)
Deletion	ACTGTCGTG ACT____TG	[3, 7]	(Empty)	GTCG
Indel b	ACTGTCGTG ACCAA_GTG	[2, 6]	CAA	TGTC

a Duplications are treated internally as a special type of insertion.

b Inversions are treated internally as a special type of indel.

### 2.3 Normalization

The normalization step generates a unique variant definition—corresponding to a HGVS identifier—for each contextual allele. This involves generating on‐the‐fly all possible HGVS expressions for the variant. A variant representation may sometimes be converted into another equivalent one by “trimming” and “shifting” it right or left by one or more base pairs without changing the resulting alternate sequence (Munz et al., 2015; Tan et al., 2015; Yen et al., 2017). A simple variant definition (after maximal “trimming”) that cannot be further shifted left is referred to as “left‐aligned”; similarly, a simple variant definition that cannot be shifted right is referred to as “right‐aligned.” If the “left‐aligned” and “right‐aligned” simple representations are the same, the variant cannot be shifted. Otherwise, by shifting the variant left and right, multiple equivalent simple variant expressions are generated, as illustrated in Table 2. In either case, the normalization step always identifies the left‐aligned simple variant definition—and the corresponding HGVS expression—for the purpose of grouping equivalent contextual alleles during canonicalization.

**Table 2:** Variants involving insertion and/or deletion and their left‐ and right‐aligned representations

Example variant	Left‐aligned	Right‐aligned
ACTG____TCGTG ACTGTAAGTCGTG	ACT____GTCGTG ACTGTAAGTCGTG	ACTGT____CGTG ACTGTAAGTCGTG
ACTGTCGTG ACTG___TG	ACTGTCGTG ACT___GTG	ACTGTCGTG ACTGT___G
AGTTCACTGCTGCTGCATCA AGTTCACTG___CTGCATCA	AGTTCACTGCTGCTGCATCA AGTTCA___CTGCTGCATCA	AGTTCACTGCTGCTGCATCA AGTTCACTGCTGC___ATCA

### 2.4 Canonicalization

Although the normalization step defines unique representation (simple left‐aligned) of contextual alleles in the context of a specific reference sequence, the canonicalization step provides a representation for nucleotide variants across multiple references (Figure 2). Because the canonical representation is by definition “context‐independent” (independent of the context of any reference sequence), it is denoted by purely conventional symbolic sign, a canonical identifier (“CAid”) and the corresponding dereferenceable URI. A single CAid denotes one or more contextual alleles. To group the contextual alleles, the canonicalization algorithm uses the reference database consisting of alignments of genomic and transcript reference sequences against the latest human genome assembly (Section 2.5). The alignments of reference sequences to GRCh38 used for canonicalization are as provided by the NCBI, EBI, and LRG. Canonical variants are not defined for amino acid sequences, as the amino acid sequences are computed on‐the‐fly from protein‐coding transcript sequences and are never aligned directly. The variant model supports the rare but unavoidable merging and splitting of canonical alleles using the concepts of “Active” and “Inactive” canonical allele, thus achieving absolute persistence of canonical allele URIs. One event triggering the merging and splitting may be the availability of a new human genome assembly where two variants that have distinct identifiers and reside in two different regions in the old assembly are merged in the new assembly. In this case, one of the two identifiers will become inactive. It is important to note that the “inactive” CAid and the corresponding URI continue to be dereferenceable. Other events that may trigger merging or splitting include some other changes in reference genome assemblies and changes in alignments.

### 2.5 Reference database

The reference database consists of alignments of reference nucleotide sequences from key genomic databases (RefSeq, ENSEMBL, and LRG) and supports the validation, normalization, and canonicalization steps. The nucleotide sequences are used as aligned against the latest human genome assembly, currently set to GRCh38. The alignments of reference sequences were imported from authoritative sources including EBI, NCBI, and the LRG consortium. To ensure high‐bandwidth operation and low‐latency response, the majority of sequences and alignments are represented as transformations—consisting of edit operations according to the imported alignments—of the latest human genome assembly, as described in detail below. This approach speeds up the mapping of alleles across different reference sequences and compresses the sequences and alignments so that the whole database can be kept in memory for fast access. To support a variety of queries, the database also includes all major reference sequence identifiers and sequence annotations such as exons, intron/exon boundaries, and coding sequences. Relations between nucleotide variants and their corresponding amino acid variants are computed on‐the‐fly.

The largest part of the reference database is the GRCh38 assembly stored as FASTA format (roughly 3 GB). Other genomic sequences, such as older assemblies (GRCh37, NCBI36), transcripts, and gene regions, are encoded as key attributes that define their alignment to the GRCh38 reference. These attributes include (1) the GRCh38 locus to which the given reference sequence is aligned, (2) A CIGAR (Concise Idiosyncratic Gapped Alignment Report) string describing the alignment, (3) list of sequences corresponding to insertions or mismatches in the CIGAR string, and (4) sequence corresponding to the unaligned part of the sequence. Rather than being directly stored, protein sequences are calculated on the fly from source transcript sequences. The whole reference database currently occupies ∼4 GB of memory.

### 2.6 Allele database

The allele database stores variant definitions and identifiers from major resources (e.g., ClinVar, dbSNP, ExAC, gnomAD). It is composed of a custom low‐level key‐value database engine with several indices that support fast querying. The database engine fulfills ACID (Atomicity, Consistency, Isolation, Durability) requirements, allowing the Registry to function as an OLTP (On‐Line Transaction Processing) system that supports real‐time registration of new variants.

### 2.7 Link database

**Figure 3:**
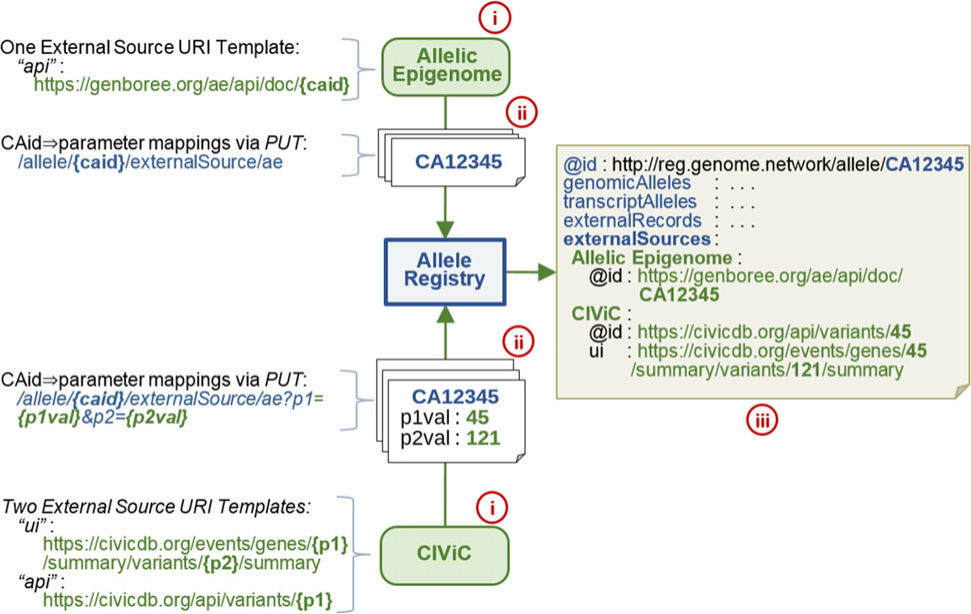
Registry API services permit on‐demand linking of variant information from external sources. **(i)** The external source indicates their RFC6570 URI template for their API and, optionally, for their UI. **(ii)** Then the external source associates one or more parameters with CAids about which they have information via PUT requests to the Registry API. Bulk uploads of associations are also supported. These parameters will be used to fill the templates, thereby creating the appropriate link. **(iii)** The Registry can now include links to these external sources in addition to its own core variant metadata. For the Allelic Epigenome case, because their API directly employs CAids, no parameter values need be supplied when registering a link via the PUT requests to the Registry. In contrast, if CIViC were to add links from Registry alleles to their data, two parameter values (*p1*, *p2*) would be registered for each CAid. Based on the CIViC templates shown, both parameter values are needed to construct the appropriate web page URL, whereas only one is needed to form the CIViC *“api”* URL

The allele database (Section 2.6) includes variant identifiers from major databases, enabling aggregation of information from these sources. In addition, the Registry provides a service to support on‐demand “layering” of additional variant information from any additional source by enabling any party to publish URI links to additional information for any subset of variants, large or small. The URIs point to the variant‐specific content that is either human readable (HTML) or machine readable (ideally RDF‐serializable JSON‐LD) or, ideally, both. The URIs are constructed on‐the‐fly using the IETF URI template (RFC 6570) specific to the external information source. The source's URI template is filled using either the variant's CAid (preferred) or the expansion values the source associated with the CAid (Figure 3). To meet the needs of large external sources, the Registry APIs support bulk upload of the associations for already registered variants and for new variants upon their registration.

### 2.8 Availability, licensing, and source code

The Allele Registry services—web app and APIs (https://reg.clinicalgenome.org)—are freely available for public use. The source code is distributed under a GNU Affero GPL v3.0 license and is available at https://github.com/BRL-BCM.

## 3 RESULTS

### 3.1 Content of Registry databases

**Figure 4:**
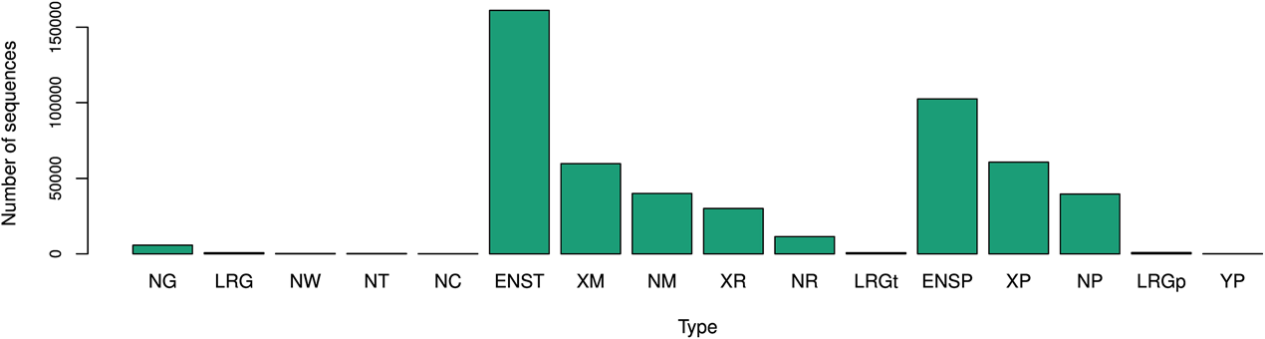
Reference sequences currently supported by the Registry. The NM, NP, and NR represent known and XM, XP, and XR represent modeled reference sequences from RefSeq (O'Leary et al., 2016). NC represents sequence of chromosomes, whereas NW, NT, and NG represent various genomic scaffolds. LRG, LRGt, and LRGp are genomic, transcript, and protein sequences from Locus Reference Genomic Database (MacArthur et al., 2014). ENST and ENSP are transcript and amino acid sequences from ENSEMBL (Aken et al., 2016)

The Registry content is stored in the reference, allele, and link databases (their implementation is described in Sections 2.5–2.7, respectively). The reference database currently contains more than 500,000 reference nucleotide and amino acid sequences (summarized in Figure 4) available from NCBI, EBI, and LRG. An update process ensures that the reference sequences, associated names and metadata, and reference alignments are kept up to date. Potential new sources of reference sequences are regularly evaluated to ensure that references from all major sources are included.

The allele database currently hosts unique Registry identifiers (CAid's) for more than 650 million distinct variants, most originally identified in major variant databases, including gnomAD, ExAC, dbSNP, MyVariant.info, COSMIC, and ClinVar (their contributions to the Registry are summarized in Table 3). Variant identifiers from these major sources are also included in the allele database, supporting querying and cross‐referencing. The database is regularly updated with new variants as they emerge in these databases. A small but growing fraction of variants comes from on‐demand registration by Registry users.

**Table 3:** Resources preregistered and cross‐linked in the ClinGen Allele Registry

Resource	Number of variants with link to source
ClinVar RCV	475,034
ClinVar Allele	590,706
ClinVar Variations	348,882
dbSNP	338,830,568
ExAC	10,175,861
gnomAD	276,797,608
myvariant.info (hg19)	339,605,025
myvariant.info (hg38)	231,910,513
COSMIC	20,581,973

The link database (Section 2.7) stores external links to those sources that have additional information about a specific variant. The Registry UI serves as a “homepage” for the variant allowing the Registry user to quickly determine if the variant in question has been reported in any of these populations, clinical and somatic cancer databases with direct links to the entry. It currently hosts more than 1.2 billion links to six sources (Table 3).

Links to ClinVar are updated biweekly. Links to other resources are updated periodically. In addition to the links imported by the ClinGen Allele Registry development team, the Registry provides a self‐service to support on‐demand “layering” of additional variant information from any additional source via URI links to additional information for any subset of variants (Sections 2.7 and 3.5).

### 3.2 Allele Registry supports multiple types of variant query

**Figure 5:**
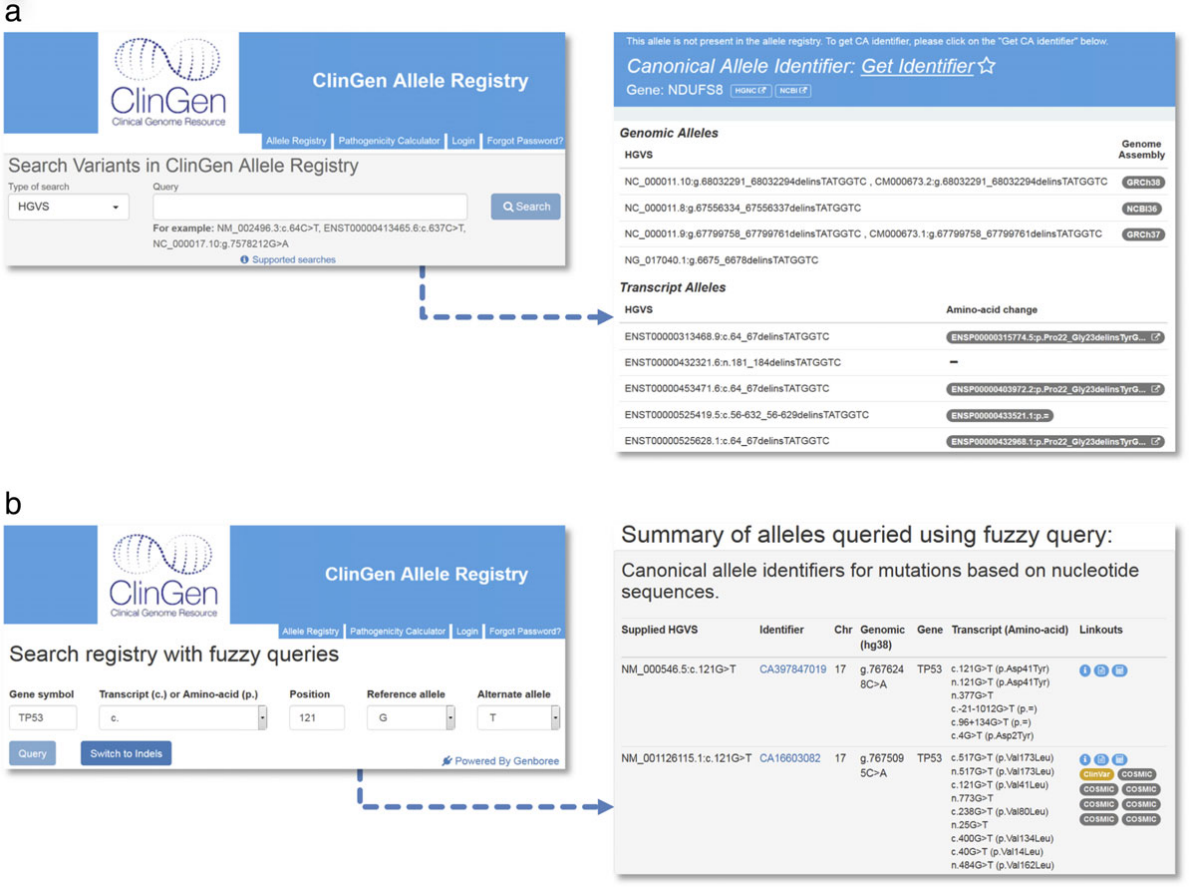
Query and registration functions accessible via the Registry web interface. (a) Example of HGVS‐based search from the Registry landing page (left) and a typical page presented to user when the variant is not registered. For logged‐in users, one click on “Get Identifier” provides canonical allele identifier. (b) Search interface for fuzzy queries where the exact transcript for which the variation is defined is not known (left). Results of example queries are shown on the right

The Registry web UI (https://reg.clinicalgenome.org) currently offers 11 variant query options, one of which (“HGVS”) is illustrated in Figure 5 **a**. Queries using a canonical allele identifier (“CAid”) or HGVS expressions return unique results, while other queries (e.g., using reference sequence and position) may result in more than one matching variant (Figure 5b and d). The web UI supports querying in individual or batch mode using a list of HGVS expressions and returns one of three responses: (1) an existing allele with CAid, (2) a valid allele that can be registered as a new allele, and (3) or an invalid allele description. The Allele Registry also supports querying by primary identifiers from multiple key sources, including ClinVar, ExAC, and dbSNP. Query results from the Registry include those primary identifiers, and thus can act as a cross‐reference service; for example, ClinVar variation identifiers may be used to locate matching variants in ExAC and vice versa.

Although widely used, HGVS expressions do not provide means for uniquely identifying a variant even in the context of the same reference sequence (Munz et al., 2015; Yen et al., 2017). Table 4 provides examples of different HGVS expressions referring to the same variant in recent publications (Yen et al., 2017). The problem is exacerbated by the growing diversity of reference sequences and prevents effective communication and aggregation of variant information from independent sources, including manual curation, text mining, and automated annotation (e.g., snpEff, VEP, and VR). The Registry addresses this problem by assigning unique CAids to sets of equivalent HGVS expressions (as illustrated in Table 4) while making the variants accessible by any valid HGVS expression.

**Table 4:** Different representations produced by different software for ground truth alleles and corresponding canonical allele identifiers

HGVS expressions	CAIDS
NM_000277.1:c.1200‐1delG NM_000277.1:c.1200delG	CA229394
NM_017739.3:c.1895+1_1895+4delGTGA NM_017739.3:c.1895+5_1895+8delGTGA	CA263965
NM_005228.3:c.2284‐6delCinsCTCCAGGA AGCCT NM_005228.3:c.2284‐5_2290dupTCCAGG AAGCCT	CA135833

Some of the current and large part of older literature include incomplete references to variants that include a gene name and partial “mutation” description without a transcript or genomic reference sequence identifier. This poses a problem for generating an HGVS expression or variant descriptions in VCF format. This incomplete allele identification is a critical issue as variant classification for rare diseases often relies on data contained in the medical literature (Richards et al., 2015). The Registry provides a web interface that helps identify and register such partially and informally defined variants. For example, for variant descriptions lacking transcript identifiers, the interface provides all transcripts available when provided a query that finds all possible transcripts given the input of a gene symbol and partial HGVS expression (Figure 5 **b**). The interface also generates variants that are not yet registered. Such variants may be immediately registered by a single click and their CAids or HGVS expressions may subsequently be used for their unambiguous identification, for example when performing variant classification.

### 3.3 The Allele Registry provides rapid and convenient access to new variant identifiers

A query for an individual variant or for multiple variants in the batch mode may identify variants that are well defined but absent from the Registry. In this case, a user has an option to register the variants (Figure 5 **b**). Although a simple query does not require a login, registration does. Registry accounts are readily available through the web interface. An interface dedicated to bulk registration of thousands of variants using as an input a list of HGVS expressions is also available. Query and registration of millions or more variants per batch are best accomplished via the Registry APIs, as we describe next.

### 3.4 Registry web API provide programmatic interoperability

The use of web APIs is the preferred interoperability method for regular and automated interactions with Registry services. The APIs are indispensable for query and registration of large number (millions) of variants: all variants, even from large resources such as dbSNP and myVariant.info can be queried or registered within 1 or 2 hr (Table 5).

**Table 5:** Summary of time required to query and number of duplicate variants identified in key variant centric resources

Source	Number of variants	Number of variants used for checking duplicates	Number of variants processed by Registry	Number of duplicates	Time for processing
dbSNP	339,334,552	19,964,466 indel	19,953,620	1,775,058	∼15 min
MyVariant.Info	412,996,966	412,996,966	412,965,634	134,881	∼90 min
ClinVar	302,036	302,036	302,024	0	40 s

The ClinGen Pathogenicity Calculator (Patel et al., 2017) and ClinGen Variant and Gene Curation Interfaces also interact with the Registry via the APIs: the Calculator accesses query and registration functionality, whereas the Curation Interfaces query the Registry using allele identifiers (CAids) to retrieve variant information relevant for curation. The Calculator registers novel variants via the API on‐the‐fly allowing its users to obtain the CAid and proceed with interpretation without delay while providing an opportunity to aggregate information generated about the same variant using different tools. ClinGen's Variant Curation Interface relies on CAids to uniquely identify a variant for curation. It supports user entry of a CAid, using the Allele Registry's API to return and save the variant and its associated information. This precise identification makes it possible for the Variant Curation interface to aggregate and display information generated about a unique variant. Thus, the Allele Registry facilitates the accurate aggregation of information for a variant for multiple tools.

To fully support Registry usage by any external application, we implemented the Registry in an API‐centric manner with Registry web UIs utilizing Registry functionality exclusively through its public HTTP REST‐APIs. Through disciplined adherence to this approach, we ensured that all the functionality accessible to human users via the web interface is also accessible programmatically via the APIs. For maximal ease of use, APIs are designed to communicate using very simple and intuitive endpoints and the response is sent back using a standard Linked Data format (RDF‐serializable JSON‐LD) or an annotated VCF file format.

### 3.5 Integration of the Registry with variant‐centric tools and databases via bidirectional links

**Figure 6:**
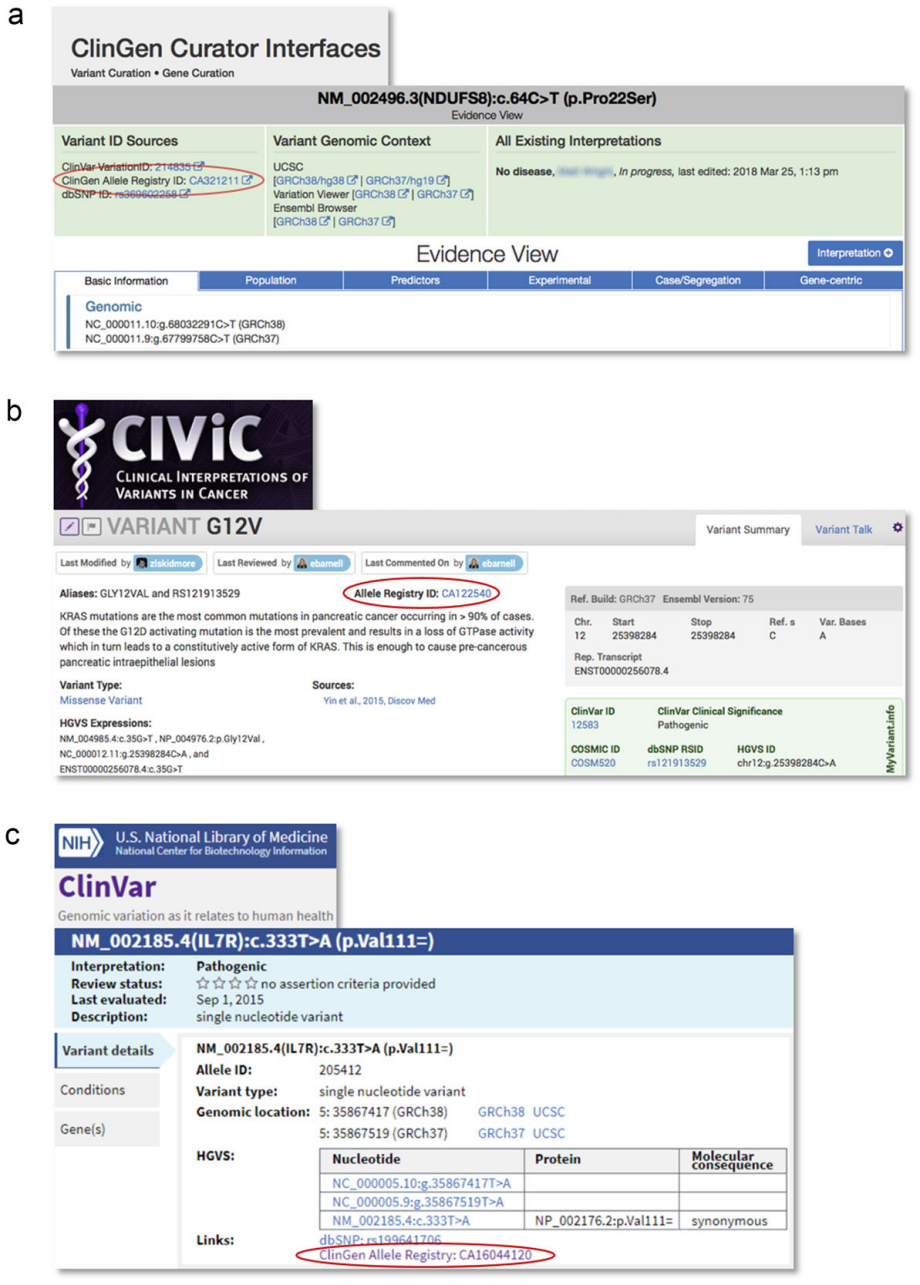
Adoption of canonical allele identifiers by variant‐centric resources. (a) ClinGen variant and gene curation interface, (b) CIViC, and (c) ClinVar. Other systems that use Allele Registry identifiers (including ClinGen Pathogenicity Calculator and Database of pathogenic variants at Keio University) are not shown for brevity

Several variant‐centric tools and databases currently interoperate with the Registry. ClinGen's Variant Curation Interface, CiVIC (Griffith et al., 2017), myVarinat.info (Xin et al., 2016), and ClinVar register their variants; store the CAids locally within their databases; and provide click‐through links to the Registry via the variant URIs embedded in their user interfaces (Figure 6). To enable linking in the other direction, Registry API services support on‐demand linking of variant information from external sources (Figure 3). Any external source may import and manage links (URIs) to variant information that is hosted at their site. This mechanism enables “layering” of additional information about registered variants by the community. The contributed links are accessible both via the Registry web UI and programmatically via the APIs. The links are generated dynamically and point to either user‐readable HTML or computer‐readable content or, preferably, both. In contrast to the human‐readable content that must be aggregated by human inspection, the machine‐readable content may be aggregated programmatically (as illustrated in Figure 3) for consumption by computer applications such as variant curation tools.

### 3.6 Variant deduplication

One “side effect” of variant registration is deduplication of variants in the registered source. The canonicalization web service may therefore be used via the APIs to deduplicate variants in both public and private databases. To demonstrate this capability at scale, we employed Allele Registry API to find duplicates in the dbSNP, ClinVar, and MyVariant.Info databases. To accomplish this, for each of these databases bulk queries with all database variants (as VCF files) were submitted using the stepwise process described in Supporting Information Sections 1.1–1.3. The Registry processed 1.3 million variants per minute for dbSNP indels, 4.6 million variants per minute for MyVariant.Info (*hg38_20171103_t4bnm6pp)*, and 450 thousand variants per minute for ClinVar. The largest percent (0.52%) of duplicate entries was found in dbSNP, a significantly lower (0.03%) in MyVariant.Info, while in ClinVar no duplicate records were found; it is consistent with the extensive curation performed by the NCBI staff of variants submitted to ClinVar before they provide a ClinVar identifier (exact numbers of duplicates in each database are in Table 5).

### 3.7 Mining linked variant information from ClinVar and ExAC to identify nucleotide variants that cause the same amino acid change while being subject to discordant pathogenicity assertions

The Registry tracks the relationship between nucleotide and amino acid variants, thus helping identify groups of nucleotide variants that have similar effects on amino acid sequences. The tracking is implemented by an optimized algorithm that efficiently calculates the effects on amino acid sequences for substitutions and small variants affecting changes in fewer than eight amino acids. To demonstrate this feature, we identified all pairs of nucleotide variants that result in the same alternate sequence with pathogenicity assertions in ClinVar (details provided in Supporting Information Section 1.4). Although there are 781 variants with consistent assertions of the that result in the same alternate sequence, we identified 65 with potentially clinically relevant discordant assertions in ClinVar (Table 6 and Supporting Information Table S1). For example, for TP53 (NM_000546.5), variants c.736AC and c.736AT both result in p.Met246Leu, however, they are interpreted as uncertain significance and likely pathogenic, respectively. This set of variants warrants re‐evaluation because variants that result in the same alternate sequence are not likely to show discordant pathogenicity in the absence of human‐specific codon bias or alteration in splicing. Similarly, in the ExAC database, we found 32 sets of variants that results in the same alternate sequence where the lowest frequency is less than 1% and the highest frequency is above 5% (a detailed description is given in Supporting Information Section 1.5), which would result in different classification evidence codes for variant classification by the ACMG/AMP criteria (Richards et al., 2015). Supporting Information Table S2 summarizes these 32 sets of variants along with their allele frequencies.

**Table 6:** Comparison of assertions in ClinVar for variants that result in identical amino acid change

	Benign	Uncertain significance	Pathogenic
Benign	70	–	–
Uncertain significance	31	140	–
Pathogenic	0	34	571

Note for simplicity the likely pathogenic and pathogenic variants were combined as well as likely benign and benign. The full list of variants and assertions is found in Supporting Information Table S1.

## 4 DISCUSSION

The ClinGen Allele Registry enables effective exchange of information about human genetic variants by providing globally unique “canonical” variant identifiers on demand. The web interfaces (web UIs) provide support for a number of key use cases, including variant query and registration, either individually or in large batches. The Registry cross‐references information about variants across major databases by collating alternate variant identifiers and supports publication and sharing of links to information about variants in any external source, large or small. The Registry is designed to scale to billions of variants and to meet the needs of all global sequencing projects and even computational prediction algorithms that output information about variants that are yet to be observed in humans or in vitro.

The Registry is accessible programmatically via well‐documented web APIs in accordance with recently articulated FAIR (“Findable, Accessible, Interoperable, Reusable”) principles (Wilkinson et al., 2016). Although the principles were originally defined with large experimental datasets in mind, they also apply more broadly to information and to the computable knowledge about subjects such as genetic variants. The ClinGen Allele Registry addresses multiple aspects of FAIRness, with an emphasis on the following two aspects of “Findability”: (a) the requirement for globally unique identifiers and (b) the requirement for rich metadata (including alternate identifiers and identifiable combinations of attributes) to facilitate search and retrieval. The Registry also implements “Accessibility” via HTTP REST‐APIs and Interoperability by providing variant information using JSON‐LD and controlled vocabularies.

One important Registry feature is the support for linking to information about registered variants in external sources. This approach to data aggregation both parallels and complements traditional data warehousing strategy (Bean & Hegde, 2016), exemplified by databases such as MyVariant.info (Xin et al., 2016) and wANNOVAR, a web server built on top of the ANNOVAR application (Chang & Wang, 2012; Wang, Li, & Hakonarson, 2010; Yang & Wang, 2015). The data warehousing strategy brings all the variant data to a single location through an “Extract‐Transform‐Load” (ETL) process. A key step in the ETL process is “deduplication” where (a) information gathered from disparate sources is being recognized as pertaining to the same entity (same variant) and can therefore be aggregated, and (b) a locally unique variant identifier (“primary key”) is assigned to index the aggregated information. This centralized strategy comes with several important limitations, including significant incremental costs associated with including each new source of variant information and the costs associated with refreshing the data from external sources via the ETL process, which often causes the information to be out of date. Rather than warehousing variant information at any single location, the Registry provides globally unique “canonical” variant URIs on demand via web (UI or API) services, thus in effect externalizing the deduplication function of traditional data warehouses. By creating a nexus for aggregation of variant conformation based on Linked Data principles and technologies, the Registry eliminates costly warehousing steps while engaging local resources at their most current state, thus accommodating better the rapidly increasing volume and diversity of variant information.

One more recent alternative to data warehousing is the distributed “hub‐and‐spoke” strategy where a centralized “hub” indexes variant information distributed across geographically distributed “nodes”, as exemplified by Leiden Open‐source Variation Database (LOVD; Fokkema et al., 2011) and GA4GH's Beacon system (Global Alliance for Genomics and Health 2016). Although showing obvious similarity to our linking approach, these systems—as currently implemented—themselves have few limitations. Specifically, LOVD imposes limits to participation, scale, and scope. Beacon has limited power to link variant information because it lacks the equivalent of the deduplication step. Although not as significant for single nucleotide variants (SNPs), it imposes significant barriers for the many complex variants encountered in clinical genetics that are either (a) SNP variants defined in the context of a multiplicity of transcript sequences or (b) non‐SNP variants such as indels that may be represented in many different ways even in the context of the same reference sequence. Either problem precludes effective aggregation of information from multiple sources about the same variant. The Registry addresses these problems by supporting indel variants and by providing canonical variant identifiers that are not dependent on variant type or sequence context.

The Registry is designed for both individual users, such as a clinician or curator using the UI to unambiguously identify a single variant found in an article or test report prior to curation, as well as a genomics pipeline that annotates and/or registers millions of variants through the provided APIs.

Finally, although registry helps to overcome several problems associated with variant identity and canonicalization, it has a few limitations in its current form. First, because the current variant model assumes that variants are identical at the genome and transcript levels, the canonicalization fails when a substitution in the genome also affects splicing, causing inclusion/exclusion of exon in the transcript, described by deletion at a transcript level and a substitution at the genomic level. Second, HGVS expressions for an indel may represent a variation that can be fractioned in the two independent indels (or indels and substitutions). This is a special case of a variant that can be described as a set of variants within a haplotype. In its present form, the registry currently does not explicitly model haplotypes and treats each distinct haplotype as a distinct variant. Finally, the registry also assumes that each variant is described at the base pair level of resolution and does not support variants such as CNVs that may not be described at that level of precision. Continuous development in coordination with key stakeholders are in process toward overcoming these limitations.

In summary, the Registry web services create an innovative nexus for effective exchange and aggregation of information about human genetic variants, thus catalyzing the emergence of a commons of variant data and knowledge required for the advancement of genome research and the genomic medicine.

## CONFLICTS OF INTEREST

SEP is a member of the Baylor Genetics Scientific Advisory Panel. AM is an employee of BCM and performs integration consulting services for BCM‐developed software including Genboree through IP Genesis, Inc. LB is employed by Sunquest Information Systems company. Sunquest is a commercial laboratory software vendor. Other authors do not have any conflicts of interest. The content is solely the responsibility of the authors and does not necessarily represent the official views of the National Institutes of Health.

## Supporting information

Supplemental Information
