# Supplementary material for: ClinGen Allele Registry links information about genetic variants
Source: Hum Mutat. 2018 Oct 11;39(11):1690–701. doi: 10.1002/humu.23637 (PMC6519371; doi:10.1002/humu.23637)
Supplement: Supplementary file 1 — Supplemental Information [file HUMU-39-1690-s001.docx]

# Supplemental Information

## Detection of duplicate variants in dbSNP

The VCF that is provided by dbSNP was downloaded from the following FTP location:

ftp://ftp.ncbi.nih.gov/snp/organisms/human_9606_b150_GRCh38p7/VCF/All_20170710.vcf.gz .

As there are multiple variants associated with single rsID, BCF tools was used to split multiallelic entries in VCF (version 1.2). We also used BCF tools to remove single nucleotide variations from the VCF file. The following commands were used to process the VCF file.

bcftools norm -m -any All_20170710.vcf.gz | bcftools view -V snps > indels.vcf

cat indels_ca.vcf | grep -v '^#' | wc -l

Output: 19964466

The resulting VCF file with indels had almost 20 million records. The VCF file was sent to the Registry for annotation by CA identifiers by POST request. The request took 13 minutes and 50 seconds to complete, including time needed to upload the VCF file on the server and download of a result file.

curl -X POST "http://reg.genome.network/annotateVcf?assembly=GRCh38&ids=CA" --data-binary @indels.vcf > indels_ca.vcf

From the annotated VCF, we filtered out and counted duplicates. We also checked the number of all records that were recognized by the Registry.

cut -f 3 indels_ca.vcf | grep -Eo "CA[0-9]{1,10}" > ca.txt

cat ca.txt | sort | uniq > unique.txt

wc -l ca.txt unique.txt

Output:

19953620 ca.txt

18178562 unique.txt

ClinGen Allele Registry was able to parse and recognize 19,953,620 of submitted variants (99.95%). Remaining 10,846 that were not processed by Registry were indels with abmbiguous base (denoted by “N” letter). The annotated VCF contains 18,178,562 unique CA ID meaning 1,775,058 of recognized indels were duplicates.

## Detection of duplicate variants in ClinVar

To detect duplicate variants in ClinVar database we used analogical approach as for dbSNP database (described in section 5.1), *i.e.*, we used CAR to annotate a VCF file published on the ClinVar webpage with CA IDs. The file was downloaded from <ftp://ftp.ncbi.nlm.nih.gov/pub/clinvar/vcf_GRCh38/clinvar_20171203.vcf.gz> . The file contains 302,036 records and the annotation process took less than 40 seconds (including downloading and uploading data).

cat clinvar_20171203.vcf | grep -v '^#' | wc -l

Output: 302036

curl -X POST "http://reg.genome.network/annotateVcf?assembly=GRCh38&ids=CA" --data-binary @clinvar_20171203.vcf >all_ca.vcf

Then we extract CA identifiers from the response and count unique and duplicate identifiers.

cut -f 3 all_ca.vcf | grep -Eo "CA[0-9]{1,9}" > ca.txt

cat ca.txt | sort | uniq > ca_uniq.txt

wc -l ca.txt ca_uniq.txt

Output:

302024 ca.txt

302024 ca_uniq.txt

CAR successfully processed 302,024 variants. Remaining 12 variants were omitted by CAR, because their sequences contain unknown base-pairs. Among recognized variants we found no duplicates; each of them was assigned to different CA ID.

## Detection of duplicate variants in MyVariant.Info

To detect duplicates in MyVariant.Info, we utilized the file *hg38_20171103_t4bnm6pp* with all MyVariant.Info identifiers based on GRCh38 reference genome. The file was downloaded from http://myvariant.info/all_ids/myvariant_hg38_ids.xz (the link was published on the page <http://biothings.io/list-of-all-hgvs-ids-in-myvariant-info/>). The file contains 412,996,966 variants identifiers and has 7.7GB. The file was divided into 4 parts. The file was also sorted locally to speed up calculations on the server side.

sort hg38_20171103_t4bnm6pp > mvi_hg38_sorted2

split -n l/4 -d mvi_hg38_sorted2

The obtained files “x00”, “x01”, “x02” and “x03” (outputs of the “split” command) were used to query Allele Registry for corresponding CA identifiers. Each query took around 21 minutes, the overall time of querying data from registry was less than 85 minutes.

curl -X POST "https://reg.genome.network/alleles.json?file=MyVariantInfo_hg38.id&fields=none+@id" -k --data-binary @x00 >x00.json

curl -X POST "https://reg.genome.network/alleles.json?file=MyVariantInfo_hg38.id&fields=none+@id" -k --data-binary @x01 >x01.json

curl -X POST "https://reg.genome.network/alleles.json?file=MyVariantInfo_hg38.id&fields=none+@id" -k --data-binary @x02 >x02.json

curl -X POST "https://reg.genome.network/alleles.json?file=MyVariantInfo_hg38.id&fields=none+@id" -k -data-binary @x03 >x03.json

Resultant CA identifiers were extracted from obtained responses and used to find duplicates.

cat x0*.json | grep '@id' | grep -Eo "CA[0-9]{1,9}" > ca.txt

sort ca.txt > ca_sort.txt

uniq ca_sort.txt > ca_uniq.txt

wc -l hg38_20171103_t4bnm6pp ca_sort.txt ca_uniq.txt

Output:

412996966 hg38_20171103_t4bnm6pp

412965634 ca_sort.txt

412830753 ca_uniq.txt

CAR was able to recognize and process 412,965,634 MyVariant.Info identifiers, what corresponds to more than 99.99% of the dataset. The recognized identifiers were assigned to different 412,830,753 CA IDs, what means that we found 134,881 duplicates. It corresponds to 0.03% of whole MyVariant.Info GRCh38 dataset.

## Identifying pairs of synonymous variants in ClinVar

In this use case, we searched for pairs of variants in ClinVar with the same effects on protein level and compared their clinical significance. As an input data we used 3 VCF files prepared in the previous example with ClinVar variants, called “clinvar_benign.vcf”, “clinvar_uncertain.vcf” and “clinvar_pathogenic.vcf”.

We employed Allele Registry to query protein effects for each set of variants.

curl ‑X POST "http://reg.genome.network/alleles.json?file=vcf&fields=none+externalRecords.ClinVarVariations.variationId+transcriptAlleles.proteinEffect.hgvsWellDefined" --data-binary @clinvar_benign.vcf > clinvar_benign.json

curl ‑X POST "http://reg.genome.network/alleles.json?file=vcf&fields=none+externalRecords.ClinVarVariations.variationId+transcriptAlleles.proteinEffect.hgvsWellDefined" --data-binary @clinvar_uncertain.vcf > clinvar_uncertain.json

curl ‑X POST "http://reg.genome.network/alleles.json?file=vcf&fields=none+externalRecords.ClinVarVariations.variationId+transcriptAlleles.proteinEffect.hgvsWellDefined" --data-binary @clinvar_pathogenic.vcf > clinvar_pathogenic.json

A simple script written in ruby was employed to compare queried variants in protein space.

./car_proteins.rb clinvar_benign.json clinvar_uncertain.json clinvar_pathogenic.json

The script “car_proteins.rb” is presented below:

| #!/usr/bin/env ruby  require 'json'  $records_all = 0  $records_parsed = 0  $records_protein = 0  def convertToData(json,data,type)  json.each { \|r\|  $records_all += 1  next if not r.key?('externalRecords')  next if not r['externalRecords'].key?('ClinVarVariations')  $records_parsed += 1  id = r['externalRecords']['ClinVarVariations'][0]['variationId'].to_i  data[id] = Hash.new  data[id]['type'] = type  data[id]['proteins'] = []  if r.key?('transcriptAlleles')  r['transcriptAlleles'].each { \|p\|  h = p["proteinEffect"]["hgvsWellDefined"]  data[id]['proteins'] << h if h =~ /^NP_/  }  end  }  end  if ARGV.size != 3  puts "Parameters:\n 1. input JSON with benign variants\n 2. ... with uncertain significant variants\n 3. ... with pathogenic variants\n"  exit  end  puts "Parse JSON files #{ARGV.inspect} ... "  benign = JSON.parse(File.read(ARGV[0]))  uncertain = JSON.parse(File.read(ARGV[1]))  pathogenic = JSON.parse(File.read(ARGV[2]))  # hash of objects (hashes):  # type = 'benign' \| 'uncertain' \| 'pathogenic'  # proteins = array of protein HGVS-es (NP_...)  data = Hash.new  puts "Parse JSON"  convertToData(benign , data, 'benign' )  convertToData(uncertain , data, 'uncertain' )  convertToData(pathogenic, data, 'pathogenic')  puts "Select protein variants"  proteins = Hash.new  data.each { \|k,v\|  v['proteins'].each { \|h\|  proteins[h] = [] if proteins[h].nil?  proteins[h] << k  }  $records_protein += 1 if v['proteins'].size > 0  }  puts "Calculating summary"  pairs = Hash.new(0)  proteins.values.each { \|v\|  v.sort!  v.size.times { \|i\|  ((i+1)...(v.size)).each { \|j\|  k1 = v[i]  k2 = v[j]  pairs[[k1,k2]] += 1  }  }  }  puts "Records - all : #{$records_all}"  puts "Records - parsed : #{$records_parsed}"  puts "Records - proteins: #{$records_protein}"  summary = Hash.new  summary['benign'] = Hash.new(0)  summary['uncertain'] = Hash.new(0)  summary['pathogenic'] = Hash.new(0)  summary_partial = Hash.new  summary_partial['benign'] = Hash.new(0)  summary_partial['uncertain'] = Hash.new(0)  summary_partial['pathogenic'] = Hash.new(0)  pairs.each { \|k,v\|  k1 = k[0]  k2 = k[1]  t1 = data[k1]['type']  t2 = data[k2]['type']  if t2 == 'benign' or t1 == 'pathogenic'  t1, t2 = t2, t1  k1, k2 = k2, k1  end  if v != data[k1]['proteins'].size or v != data[k2]['proteins'].size  next # not all protein effects are the same  else  summary[t1][t2] += 1  if t1 != t2  puts "Conflict #{t1}-#{t2}: #{k1} #{k2}"  end  end  }  puts "All proteins effects are the same:"  summary.each { \|k1,v1\|  v1.each { \|k2,v2\|  puts "#{k1}-#{k2}:\t#{v2}"  }  } |
| --- |

## Identifying synonymous variants in ExAC

In this use case, we searched for groups of variants in ExAC with the same effects on protein level and compared their population frequencies. As an input data we used VCF file downloaded from <ftp://ftp.broadinstitute.org/pub/ExAC_release/release1/ExAC.r1.sites.vep.vcf.gz> . The file was preprocessed and used for querying from Allele Registry protein effects and corresponding links to MyVariant.Info. Then we searched for groups of variants with the same protein effect and queried MyVariant.Info for population frequencies reported by ExAC. Finally, we built a table with all groups of synonymous variants that contain at the same time at least one frequency below 1% and at least one frequency above 5%. The final summary is presented in Table S3.

Below we present a whole procedure employed for processing ExAC data. First, BCF tool (version 1.2) was used to split multiallelic entries in original VCF file. Obtained file was additionally truncated and his header was save in a new file “exac_chrs.txt”.

bcftools norm -m -any ExAC.r1.sites.vep.vcf > exac_single.vcf

cat exac_single.vcf | cut -d \= -f 1 > exac_single_reduced.vcf

cat exac_single_reduced.vcf | grep '^#' | grep -v '^##contig=<ID=[^1-9XYM]' > exac_chrs.vcf

Then the header-only file “exac_chrs.vcf” was edited by hand and the line:

##assembly=GRCh37

was added as a second line in the file. In the next step, records with variants were appended to the file before submitting it to the Registry.

cat exac_single_reduced.vcf | grep '^[1-9XYM]' >> exac_chrs.vcf

curl -X POST "http://reg.genome.network/alleles.json?file=vcf&fields=none+@id+externalRecords.MyVariantInfo_hg19.id+transcriptAlleles.proteinEffect.hgvsWellDefined" --data-binary @exac_chrs.vcf > exac.json

The obtained response was filtered and processed by home-made ruby scripts to produce the output table.

cat exac.json | grep MyVariantInfo_hg19 | grep hgvsWellDefined | grep NP_ > exac_filtered.json

./car_filter.rb exac_filtered.json to_check.txt

./mvi_freq.rb to_check.txt > out.txt

The script “car_filter.rb” is presented below:

| #!/usr/bin/env ruby  require 'json'  if ARGV.size != 2  puts "Parameters:\n 1. JSON file with variants\n 2. output file\n"  exit  end  data = Hash.new  current_chr = ''  hg38id = Hash.new(0)  out = File.open(ARGV[1], 'w')  File.open(ARGV[0], "r").each_line do \|line\|  r = JSON.parse(line[1..-1])  caid = r['@id'].split('CA')[-1].to_i  hg38id[caid] += 1  next if hg38id[caid] > 1  id = r["externalRecords"]["MyVariantInfo_hg19"][0]['id']  chr = id.split(':')[0]  if chr != current_chr  data.each { \|k,v\|  if v.size > 2  out.puts "#{v.to_json}"  end  }  data = Hash.new  current_chr = chr  end  pe = []  r['transcriptAlleles'].each { \|p\|  h = p["proteinEffect"]["hgvsWellDefined"]  pe << h if h =~ /^NP_/  }  data[pe] = [] if data[pe].nil?  data[pe] << id  end  out.close |
| --- |

The script “mvi_freq.rb” looks like follows:

| #!/usr/bin/env ruby  require 'json'  require 'uri'  if ARGV.size != 1  puts "Parameters:\n 1. input file"  exit  end  File.open(ARGV[0], "r").each_line do \|line\|  line.strip!  r = JSON.parse(line)  x = r.join(',')  url = "http://myvariant.info/v1/query?q=#{x}"  url << "&assembly=hg19&fields=exac.af,exac.ac,exac.an&scopes=_id"  response = `curl -X POST \"#{url}\"`  freq = []  JSON.parse(response).each { \|y\|  if y['exac'] and y['exac']['ac'] and y['exac']['an']  ac = y['exac']['ac']['ac_adj'] * 1.0  an = y['exac']['an']['an_adj']  freq << [ac/an,y['_id']]  end  }  freq.sort!  next if freq.size < 2  if freq[0][0] < 0.01 and freq[-1][0] > 0.05  effects = []  url = "http://reg.genome.network/alleles?MyVariantInfo_hg19.id="  url << "#{URI::encode(freq[0][1])}&fields="  url << "none+transcriptAlleles.proteinEffect.hgvsWellDefined"  response = `curl -X GET \"#{url}\"`  JSON.parse(response)[0]['transcriptAlleles'].each { \|t\|  h = t["proteinEffect"]["hgvsWellDefined"]  effects << h if h =~ /^NP_/  }  puts "#{effects.join(', ')}"  freq.each { \|f\|  url = "http://reg.genome.network/alleles?MyVariantInfo_hg19.id="  url << "#{URI::encode(f[1])}&fields="  url << "#{URI::encode("none+@id+externalRecords")}"  response = `curl -X GET \"#{url}\"`  rj = JSON.parse(response)[0]  exac_id = rj['externalRecords']['ExAC'][0]['id']  puts "#{f[0]}\t#{f[1]}\t#{exac_id}\t#{rj['@id'].split('/')[-1]}"  }  end  end |
| --- |

**Table S1. Synonymous nucleotide variants with discordant interpretations in ClinVar.**

| **ClinVar variations** | **Interpretations** |
| --- | --- |
| 8524:393446 | Benign/Likely Benign:VUS |
| 294920:73491 | Benign/Likely Benign:VUS |
| 193142:193135 | Benign/Likely Benign:VUS |
| 334444:286350 | Benign/Likely Benign:VUS |
| 233063:232637 | Benign/Likely Benign:VUS |
| 44970:199930 | Benign/Likely Benign:VUS |
| 179202:199930 | Benign/Likely Benign:VUS |
| 261169:425370 | Benign/Likely Benign:VUS |
| 163130:238901 | Benign/Likely Benign:VUS |
| 95608:197812 | Benign/Likely Benign:VUS |
| 95608:197813 | Benign/Likely Benign:VUS |
| 232551:411760 | Benign/Likely Benign:VUS |
| 262802:410997 | Benign/Likely Benign:VUS |
| 221954:366953 | Benign/Likely Benign:VUS |
| 300087:300088 | Benign/Likely Benign:VUS |
| 419028:418176 | Benign/Likely Benign:VUS |
| 130344:300363 | Benign/Likely Benign:VUS |
| 412842:236957 | Benign/Likely Benign:VUS |
| 125973:142616 | Benign/Likely Benign:VUS |
| 413741:135377 | Benign/Likely Benign:VUS |
| 180306:318955 | Benign/Likely Benign:VUS |
| 142040:406646 | Benign/Likely Benign:VUS |
| 54258:54260 | Benign/Likely Benign:VUS |
| 323794:288484 | Benign/Likely Benign:VUS |
| 260640:283266 | Benign/Likely Benign:VUS |
| 422030:422029 | Benign/Likely Benign:VUS |
| 260867:339213 | Benign/Likely Benign:VUS |
| 205024:205048 | Benign/Likely Benign:VUS |
| 93137:128497 | Benign/Likely Benign:VUS |
| 193325:95305 | Benign/Likely Benign:VUS |
| 143412:143390 | Benign/Likely Benign:VUS |
| 179669:444011 | VUS:Pathogenic/Likely Pathogenic |
| 48045:200946 | VUS:Pathogenic/Likely Pathogenic |
| 195490:217224 | VUS:Pathogenic/Likely Pathogenic |
| 167648:378898 | VUS:Pathogenic/Likely Pathogenic |
| 90098:237334 | VUS:Pathogenic/Likely Pathogenic |
| 90099:237334 | VUS:Pathogenic/Likely Pathogenic |
| 410320:430216 | VUS:Pathogenic/Likely Pathogenic |
| 284365:265409 | VUS:Pathogenic/Likely Pathogenic |
| 205551:432901 | VUS:Pathogenic/Likely Pathogenic |
| 45221:45220 | VUS:Pathogenic/Likely Pathogenic |
| 45228:376275 | VUS:Pathogenic/Likely Pathogenic |
| 45278:45279 | VUS:Pathogenic/Likely Pathogenic |
| 24893:38596 | VUS:Pathogenic/Likely Pathogenic |
| 24888:38597 | VUS:Pathogenic/Likely Pathogenic |
| 24888:38620 | VUS:Pathogenic/Likely Pathogenic |
| 428247:440220 | VUS:Pathogenic/Likely Pathogenic |
| 411298:426015 | VUS:Pathogenic/Likely Pathogenic |
| 409541:429208 | VUS:Pathogenic/Likely Pathogenic |
| 393205:181304 | VUS:Pathogenic/Likely Pathogenic |
| 216571:217271 | VUS:Pathogenic/Likely Pathogenic |
| 406568:376587 | VUS:Pathogenic/Likely Pathogenic |
| 406586:186737 | VUS:Pathogenic/Likely Pathogenic |
| 406596:376670 | VUS:Pathogenic/Likely Pathogenic |
| 142562:12371 | VUS:Pathogenic/Likely Pathogenic |
| 44987:44988 | VUS:Pathogenic/Likely Pathogenic |
| 44987:44989 | VUS:Pathogenic/Likely Pathogenic |
| 55417:55414 | VUS:Pathogenic/Likely Pathogenic |
| 267219:267221 | VUS:Pathogenic/Likely Pathogenic |
| 208273:93213 | VUS:Pathogenic/Likely Pathogenic |
| 432270:9189 | VUS:Pathogenic/Likely Pathogenic |
| 43396:181591 | VUS:Pathogenic/Likely Pathogenic |
| 215340:215339 | VUS:Pathogenic/Likely Pathogenic |
| 408903:206331 | VUS:Pathogenic/Likely Pathogenic |
| 411872:29955 | VUS:Pathogenic/Likely Pathogenic |

**Table S2**: Synonymous variants in ExAC with markedly different frequency, grouped by effects on proteins.

| **Frequencies** | **ExAC identifiers** | **CAR identifiers** |
| --- | --- | --- |
| *NP_277071.2:p.Lys115_Glu116dup, NP_001300825.1:p.Lys115_Glu116dup,*  *NP_001300911.1:p.Lys115_Glu116dup, NP_076916.2:p.Lys125_Glu126dup* | | |
| 0.000008 | 1-1647895-T-TTCTTTC | CA530586 |
| 0.000008 | 1-1647893-C-CTCTCTT | CA530583 |
| 0.497361 | 1-1647893-C-CTTTCTT | CA530582 |
| *NP_001156365.1:p.Lys75dup, NP_001156366.1:p.Lys75dup, NP_001156367.1:p.Lys75dup, NP_055471.2:p.Lys75dup* | | |
| 0.000053 | 1-175129943-C-CTTT | CA1255091 |
| 0.000068 | 1-175129946-C-CTTT | CA1255097 |
| 0.185287 | 1-175129924-C-CCTT | CA1255079 |
| *NP_079466.3:p.Tyr905Ter* | | |
| 0.000041 | 2-98129733-A-T | CA1788565 |
| 0.000079 | 2-98129730-CCTA-C | CA1788560 |
| 0.243844 | 2-98129733-A-C | CA1788564 |
| *NP_060493.3:p.Gly814del* | | |
| 0.000008 | 3-183493742-CCGG-C | CA2722541 |
| 0.000008 | 3-183493770-AGGC-A | CA2722560 |
| 0.448473 | 3-183493743-CGGA-C | CA2722543 |
| *NP_060876.5:p.Thr113_Ala114insValThrGlnGluThr* | | |
| 0.000017 | 3-195518115-C-CTCCTGCGTAACAGTT | CA2775518 |
| 0.000050 | 3-195518112-T-TGTCTCCTGTGTAACA | CA2775515 |
| 0.752748 | 3-195518112-T-TGTCTCCTGCGTAACA | CA2775514 |
| *NP_055023.2:p.Ser1050_Asp1055del* | | |
| 0.000388 | 4-88536955-TAGCAGTGACAGCAGTGAC-T | CA3001436 |
| 0.008425 | 4-88536952-CGATAGCAGTGACAGCAGT-C | CA3001431 |
| 0.236234 | 4-88536901-CAGCAGTGACAGCAGCGAT-C | CA3001403 |
| *NP_055023.2:p.Ser1173_Asp1178dup* | | |
| 0.000050 | 4-88537267-C-CGACAGCAGCGATAGCAGT | CA3001713 |
| 0.001056 | 4-88537261-A-AAGCAGTGACAGCAGCGAT | CA3001710 |
| 0.001851 | 4-88537306-T-TAGCAGCGACAGCAGTGAC | CA3001748 |
| 0.223300 | 4-88537261-A-AAGCAGCGACAGCAGCGAT | CA3001706 |
| *NP_001078846.1:p.Gly21dup* | | |
| 0.000017 | 5-112824064-G-GCCA | CA3371242 |
| 0.000019 | 5-112824067-G-GCCA | CA3371247 |
| 0.179965 | 5-112824048-T-TGCC | CA3371227 |
| *NP_115927.1:p.Glu771del* | | |
| 0.000043 | 6-43250749-AGAG-A | CA3819840 |
| 0.000076 | 6-43250734-AGAG-A | CA3819829 |
| 0.000264 | 6-43250764-AGAG-A | CA3819848 |
| 0.000306 | 6-43250779-AGAG-A | CA3819853 |
| 0.215704 | 6-43250725-GGAA-G | CA3819822 |
| *NP_003185.1:p.Gln95del, NP_001165556.1:p.Gln75del* | | |
| 0.000017 | 6-170871004-GCAA-G | CA4108272 |
| 0.000267 | 6-170871046-ACAG-A | CA4108303 |
| 0.053691 | 6-170871037-GCAA-G | CA4108293 |
| *NP_001091089.1:p.Gly392dup* | | |
| 0.000037 | 7-1586657-G-GCCA | CA4121362 |
| 0.000292 | 7-1586654-G-GCCA | CA4121359 |
| 0.000572 | 7-1586660-G-GCCA | CA4121363 |
| 0.549409 | 7-1586653-A-AGCC | CA4121357 |
| *NP_005388.2:p.Ser30_Pro31dup, NP_001018121.1:p.Ser30_Pro31dup* | | |
| 0.000000 | 7-131241050-C-CGACGGT | CA4488797 |
| 0.000033 | 7-131241053-C-CGGCGAT | CA4488798 |
| 0.257272 | 7-131241029-G-GGGCGAC | CA4488788 |
| *NP_981948.1:p.Gly54_Gly55dup* | | |
| 0.000350 | 9-12775858-T-TGGCGGC | CA4985718 |
| 0.057631 | 9-12775861-T-TGGCGGC | CA4985721 |
| *NP_001004137.1:p.Ser305Arg* | | |
| 0.000008 | 11-4567333-A-C | CA5832852 |
| 0.000008 | 11-4567335-C-A | CA5832854 |
| 0.392172 | 11-4567335-C-G | CA5832853 |
| *NP_703145.1:p.Leu35_Ser36insCysIleTyrLeuValThrIle* | | |
| 0.000008 | 11-7818383-G-GATATGGTTACAAGGTAGATGC | CA5869084 |
| 0.000008 | 11-7818383-G-GATATGGTTACTAGGTAGATGC | CA5869082 |
| 0.000067 | 11-7818383-G-GATATGGTTACCAAGTAGATGC | CA5869081 |
| 0.246396 | 11-7818383-G-GATATGGTTACCAGGTAGATGC | CA5869080 |
| *NP_001138408.1:p.Glu200del* | | |
| 0.000237 | 11-63533328-CTCT-C | CA6064823 |
| 0.090290 | 11-63533334-TTCC-T | CA6064825 |
| *NP_001035009.1:p.Gln400del, NP_001129206.1:p.Gln516del, NP_597733.2:p.Gln455del* | | |
| 0.000000 | 12-6777111-CTGT-C | CA6413763 |
| 0.000168 | 12-6777066-CTGT-C | CA6413745 |
| 0.569505 | 12-6777069-TTGC-T | CA6413747 |
| *NP_000414.2:p.Ser100_Ser101insGlyPheGlyGlyGly* | | |
| 0.000022 | 12-53045627-G-GCCGCCTCCAAAGCCA | CA6585894 |
| 0.000034 | 12-53045626-T-TGCCACCTCCAAAGCC | CA6585891 |
| 0.209499 | 12-53045626-T-TGCCGCCTCCAAAGCC | CA6585889 |
| *NP_031376.3:p.Gln204del* | | |
| 0.000026 | 12-76424910-CTGT-C | CA6693459 |
| 0.988517 | 12-76424937-TTGC-T | CA6693480 |
| *NP_078772.1:p.Gln127del* | | |
| 0.000758 | 14-77493755-CTGT-C | CA7283969 |
| 0.003955 | 14-77493812-CTGT-C | CA7284030 |
| 0.024694 | 14-77493761-TTGC-T | CA7283975 |
| 0.365193 | 14-77493791-CTGT-C | CA7284010 |
| *NP_078772.1:p.Gln126_Gln127del* | | |
| 0.000641 | 14-77493794-TTGCTGC-T | CA7284018 |
| 0.001877 | 14-77493809-CTGCTGT-C | CA7284026 |
| 0.009180 | 14-77493788-CTGCTGT-C | CA7284004 |
| 0.403546 | 14-77493761-TTGCTGC-T | CA7283973 |
| *NP_065190.2:p.Gln132del* | | |
| 0.000009 | 16-67876793-GCAA-G | CA8118188 |
| 0.000303 | 16-67876820-GCAA-G | CA8118223 |
| 0.000915 | 16-67876802-GCAA-G | CA8118201 |
| 0.001176 | 16-67876778-ACAG-A | CA8118169 |
| 0.322394 | 16-67876823-ACAG-A | CA8118228 |
| *NP_055576.2:p.Pro238_Met239del, NP_001257908.1:p.Pro90_Met91del, NP_001257907.1:p.Pro90_Met91del, NP_001257906.1:p.Pro238_Met239del, NP_001257905.1:p.Pro251_Met252del, NP_001257904.1:p.Pro238_Met239del* | | |
| 0.000033 | 16-71956506-GTGCCAA-G | CA8157429 |
| 0.014119 | 16-71956529-CATGCCT-C | CA8157447 |
| 0.337196 | 16-71956511-AATGCCC-A | CA8157432 |
| *NP_000826.2:p.Glu1048_Leu1049insProProGlu* | | |
| 0.000000 | 17-72839135-C-CGGGGGCTCA | CA8751998 |
| 0.004464 | 17-72839130-A-AGCTCCGGTG | CA8751995 |
| 0.785714 | 17-72839130-A-AGCTCCGGGG | CA8751994 |
| *NP_003646.2:p.Ala509_Ala510dup* | | |
| 0.000019 | 17-77807926-C-CGCCGCT | CA8812006 |
| 0.000054 | 17-77807910-G-GTGCCGC | CA8811993 |
| 0.088966 | 17-77807917-T-TGCCGCC | CA8811995 |
| *NP_001276908.1:p.Gln405del, NP_001276909.1:p.Gln405del, NP_004747.1:p.Gln446del* | | |
| 0.000035 | 19-41173901-CTGT-C | CA9449559 |
| 0.001648 | 19-41173874-TTGC-T | CA9449533 |
| 0.004439 | 19-41173904-TTGC-T | CA9449562 |
| 0.078101 | 19-41173895-CTGT-C | CA9449550 |
| *NP_001276908.1:p.Gln403_Gln405del, NP_001276909.1:p.Gln403_Gln405del, NP_004747.1:p.Gln444_Gln446del* | | |
| 0.000127 | 19-41173874-TTGCTGCTGC-T | CA9449534 |
| 0.001976 | 19-41173889-CTGCTGCTGT-C | CA9449546 |
| 0.067578 | 19-41173898-TTGCTGTTGC-T | CA9449552 |
| *NP_001276908.1:p.Gln404_Gln405del, NP_001276909.1:p.Gln404_Gln405del, NP_004747.1:p.Gln445_Gln446del* | | |
| 0.000193 | 19-41173904-TTGCTGC-T | CA9449564 |
| 0.000380 | 19-41173874-TTGCTGC-T | CA9449535 |
| 0.644749 | 19-41173892-CTGCTGT-C | CA9449548 |
| *NP_002143.1:p.Asp261dup* | | |
| 0.000009 | 19-49657748-A-ATCG | CA9568406 |
| 0.000026 | 19-49657751-A-ATCG | CA9568409 |
| 0.060154 | 19-49657710-A-ACAT | CA9568375 |
| *NP_690051.1:p.Gly836_Pro837dup, NP_006038.2:p.Gly836_Pro837dup,*  *NP_001185769.1:p.Gly836_Pro837dup, NP_001185767.1:p.Gly836_Pro837dup* | | |
| 0.000068 | 20-34240733-T-TTGGGCC | CA9836513 |
| 0.004289 | 20-34240734-T-TGGGCCA | CA9836516 |
| 0.055967 | 20-34240740-A-AGGGCCG | CA9836524 |
| *NP_859016.1:p.Cys105_Val109del* | | |
| 0.000009 | 21-46057634-TGTGCCCGTCTGCTGC-T | CA10060491 |
| 0.191841 | 21-46057625-TGTCTGCTGTGTGCCC-T | CA10060482 |
| *NP_002421.3:p.Gln550del* | | |
| 0.000178 | 22-28194909-CTGT-C | CA10166365 |
| 0.000234 | 22-28194930-CTGT-C | CA10166382 |
| 0.003812 | 22-28194880-CGCT-C | CA10166350 |
| 0.059528 | 22-28194933-TTGC-T | CA10166384 |
